# War, Pestilence, and Famine

**Published:** 1899-09-23

**Authors:** 


### The Hospital

A Journal of

The Medical Sciences and Hospital Administration.

Vol. XXVI.—No. 678.] Edited by Sir Henry Burdett, K.C.B., and Solomon C. Smith, M.D., M.R.C.P. [September 23, 1899.

### WAR, PESTILENCE, AND FAMINE.

Recent news from India gives ground for hoping that the fear of famine which a few weeks ago stood as a gaunt spectre before a large number of our Indian fellow subjects is passing away, for another year at least. The clouds of war and of pestilence, however, appear to be gathering ever more ominously around, and if we consider the circumstances of the present in the light of the history of the past; if we remember how deeply one of the greatest of pestilences has taken root in the country from which it is proposed to dispatch troops to take part in the war that seems to be so nearly within sight; and if we recall how persistently this disease has clung to the very port from which it is likely that these troops, these war materials, and, more dangerous still, these camp followers will be dispatched, we see plenty of cause for fearing that the old association between war and pestilence which has been so often observed that it has got even into our Litany, will receive yet another illustration before many months have gone by. The fact that the infection of plague has now been present in Egypt for a considerable time, and has not yet given rise to any widespread outbreak of the disease, and the further fact that on reaching Portugal, although the malady took root, it has so far failed to attack more than a very small proportion of the population, have led hopeful people in this country to look with much complacency on the future; and, indeed, the well-fed, well-groomed, well-housed Englishman has good grounds for the confidence in his own immunity which he feels. But England is not all the world, and even if, in this matter, we have somewhat selfishly refrained from troubling about those who are not our fellow-subjects, we now must feel that the matter touches ourselves, for one of the terrors which hangs over this coming war of ours is pestilence.

Unfortunately, we are to a large extent in the dark as to the causes which now and again lead such diseases as cholera and plague to spread beyond their natural confines, to sweep across continents and round the globe, and, as the saying is, to become pandemic. But it has seemed to careful observers that these widespread outbursts have often been associated with great movements of population, with great religious festivals, with great fairs, and with war, and the history of the present plague prevalence, showing as it does that the disease already displays an abnormal virulence and tendency to spread, points strongly to the probability of its spreading still more widely should the outbreak of war lead to that massing of men and that marching of armies by which such a disease is apt to be disseminated.

Until five years ago, so far as the mass of mankind was concerned, plague was a matter of merely bookish interest. It was known to have prevailed extensively in Europe, coming and going with great variability during several centuries. It was known again that towards the end of the 17th century it had disappeared from the face of Western Europe in the course of a very few years. It was, of course, well known also that in certain parts of Central Asia there was still almost always more or less plague going on, but the steady recession of the disease into its Asian fastnesses made us all confident that it was gone from us for good.

Five years ago plague broke out in Hong Kong. This was by no means the first step in its progress, but it was the first that came to public notice. Then it spread through Southern China, and after two years appeared in Bombay, having thus travelled 3,000 miles westward. On arriving in Bombay, and thus touching a railway system, its progress was rapid, and in six months not only had it caused a great mortality in that city, but it had spread to other important centres in the Presidency; and ever since then India has suffered greatly, the disease spreading to new districts, as well as cropping up afresh, time after time, in places that it had seemed to have deserted. In some towns the mortality has been enormous; in Poona a few weeks ago the inhabitants were dying at the rate of 150 per day, a rate which may be understood from a statement recently made by Professor Simpson, who said that "if a similar mortality prevailed in London the metropolis would lose over 10,000 persons a day." There is then no sign of decadence of plague in India as yet. But not content with India, and undeterred by all the restrictive measures opposed to its progress, plague has continued to spread. It has, again quoting Professor Simpson, "reached the Persian Gulf, Penang, the Mauritius, the French island of Reunion, Madagascar, Jedda, Egypt, and if rumour is correct, the Gold Coast of the French possessions in West Africa, and now Portugal," and we may add to the above list the outbreak in Astrakan. What is quite certain is that plague is at present showing the strongest possible tendency to become pandemic. What it wants now, to help it on, is just a fillip—a big war, a great movement of men. Will it get this fillip? and if it does, what will be the result? In the present temper of plague the transport of an army from a plague-stricken part of India, with all the tag-rag-and-bob-tail of followers with which an army fresh from India loves to move, is a very serious matter.

